# Predictors of graft signal-to-noise ratio after medial meniscus reconstruction using tendon autograft

**DOI:** 10.1097/MD.0000000000048721

**Published:** 2026-05-08

**Authors:** Tian-Wang Zhu, Wan-Rui Shi, Hao Chen, Chun-Hui Li, Rui-Xin Li, Yan-Lin Li

**Affiliations:** a Department of Sports Medicine, The First Affiliated Hospital of Kunming Medical University, Kunming Medical University, Kunming, China; b Spinal Surgery Department, Orthopedic Department, Dalian University Affiliated Xinhua Hospital, Dalian University, Dalian, China; c Sports Medicine Department, Dalian University Affiliated Xinhua Hospital, Dalian University, Dalian, China.

**Keywords:** correlation, meniscus reconstruction using tendon autograft, meniscus replacement, ordinary least squares linear regression, Pearson correlation, predictor, signal-to-noise ratio (SNR)

## Abstract

Although meniscus reconstruction using tendon autograft is a promising technique, its indications, contraindications, optimal surgical technique, and outcomes remain unclear. This study aimed to identify predictors of graft signal-to-noise ratios (SNRs) on magnetic resonance images after medial meniscus reconstruction using tendon autograft. This study selected 11 patients (36.000 ± 4.733 years; 7 men and 4 women) who underwent medial meniscus reconstruction using tendon autograft. Correlation and regression analyses were used to evaluate the associations between the graft SNRs and potential factors. The midbody SNR at 1 year postoperatively was correlated with the body mass index (*P* = .013) and bilateral hip–knee–ankle angle difference (*P* = .026). The anterior horn SNR at 1 year postoperatively was significantly correlated with the absolute joint space width (*P* = .003) and remaining anterior horn cross-sectional area (*P* = .012). The posterior horn SNR at 1 year postoperatively was significantly correlated with the absolute joint space width (*P* = .042). The anterior intratunnel SNR at 1 year postoperatively was significantly correlated with the Kellgren–Lawrence grade (*P* = .006), hip–knee–ankle angle (*P* = .030), bilateral hip–knee–ankle angle difference (*P* < .001), relative joint space width (*P* = .037), and bilateral medial edge incline angle difference (*P* = .031). The posterior intratunnel SNR at 1 year postoperatively was significantly correlated with the Kellgren–Lawrence grade (*P* = .019), hip–knee–ankle angle (*P* = .039), bilateral hip–knee–ankle angle difference (*P* = .001), relative joint space width (*P* = .034), and bilateral medial edge incline angle difference (*P* = .011). The mean SNR at 1 year postoperatively was significantly correlated with the Kellgren–Lawrence grade (*P* = .019), hip–knee–ankle angle (*P* = .014), bilateral hip–knee–ankle angle difference (*P* = .005), absolute joint space width (*P* = .003), relative joint space width (*P* = .045), and bilateral medial edge incline angle difference (*P* = .009). The preoperative absolute joint space width, bilateral hip–knee–ankle angle difference, body mass index, and remaining anterior horn cross-sectional area at 1 week postoperatively were significant predictors of graft SNRs at 1 year postoperatively. These preliminary findings on graft remodeling may help to improve patient selection and surgical technique for meniscus reconstruction using tendon autograft.

## Graphical abstract

**Figure:**
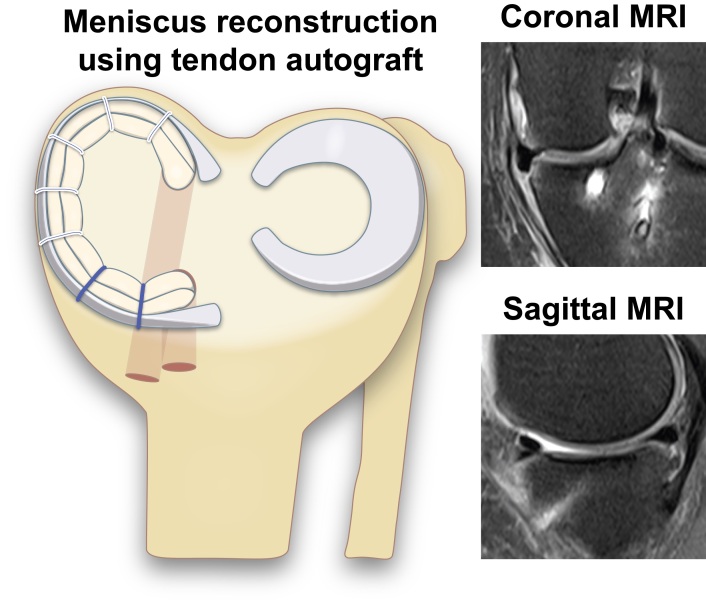

## 1. Introduction

The meniscus conforms to the space between the tibia and femur. It distributes and cushions compressive forces between the tibia and femur and stabilizes the knee joint.^[1]^ Meniscus injuries are common sports injuries. For unrepairable meniscus injuries, meniscectomy is often performed to avoid injury expansion, relieve pain, and address joint locking. After meniscectomy, the onset and progression of osteoarthritis are usually inevitable.^[2]^ To restore the function of the meniscus and to prevent osteoarthritis, meniscus replacement is recommended for patients too young for joint replacement.^[3]^ Meniscus allograft transplantation and artificial meniscal scaffolds have been used to replace the meniscus. However, their low availability, high cost, and limited survival rate limit their application.^[4–8]^ In regions where meniscus allografts and artificial meniscal scaffolds are unavailable, meniscus reconstruction using tendon autograft may be the best choice for meniscus replacement. Studies found that meniscus reconstruction using tendon autograft protects articular cartilage, relieves pain, and improves quality of life.^[9–12]^ Although meniscus reconstruction using tendon autograft is a promising technique, its indications, contraindications, optimal surgical technique, and outcomes remain unclear because of limited evidence.^[13]^

Understanding graft remodeling requires insight into its composition. Histological sections and staining can be used to analyze it.^[9]^ However, this method is not suitable for routine postoperative evaluation in clinical practice because of its invasive nature. In contrast, magnetic resonance imaging (MRI) is noninvasive. Under specific imaging parameters, tissue signal intensity reflects its composition, including proton (^1^H) density. The signal-to-noise ratio (SNR), a more standardized and comparable quantitative measure, is used to assess graft remodeling and graft maturity after ligament reconstruction, including anterior cruciate ligament reconstruction, posterior cruciate ligament reconstruction, and lateral ankle ligament reconstruction.^[14–16]^ However, to our knowledge, no study has examined graft signal intensity or graft SNR after meniscus reconstruction using tendon autograft.

This study aimed to identify predictors of graft SNRs on magnetic resonance (MR) images after meniscus reconstruction using tendon autograft. These results may enable surgeons to predict graft SNRs based on routine demographic information and images obtained in clinical practice. The hypothesis is that the physical and biological environment of the graft, as suggested by demographic information and imaging measurements, may influence graft remodeling.

## 2. Methods

### 2.1. Ethics

This retrospective cohort study was approved by the Ethics Committee of Dalian University Affiliated Xinhua Hospital (Number: 2024-43-02).

### 2.2. Patient selection

This study included 11 patients who underwent medial meniscus reconstruction using tendon autograft at the Sports Medicine Department, Dalian University Affiliated Xinhua Hospital between July 2021 and November 2024. The inclusion criteria were as follows: having undergone medial meniscus reconstruction using tendon autograft; availability of complete demographic information; availability of preoperative anteroposterior knee, anteroposterior lower limb, and lateral tibial radiographs; and availability of postoperative knee MR images acquired at 1 week and 1 year. The exclusion criterion was the integrity, resolution, contrast, or position of the bone or graft on radiographs or MR images that precluded accurate measurement. Indications for surgery included meniscus deficiency, pain, and age <50 years. We strictly limited age because it may influence graft remodeling.^[17]^ Contraindications for surgery included irreparable chondral injury, ligamentous laxity, arthrofibrosis, or active infection.^[18,19]^

### 2.3. Surgery

**Figure 1.**
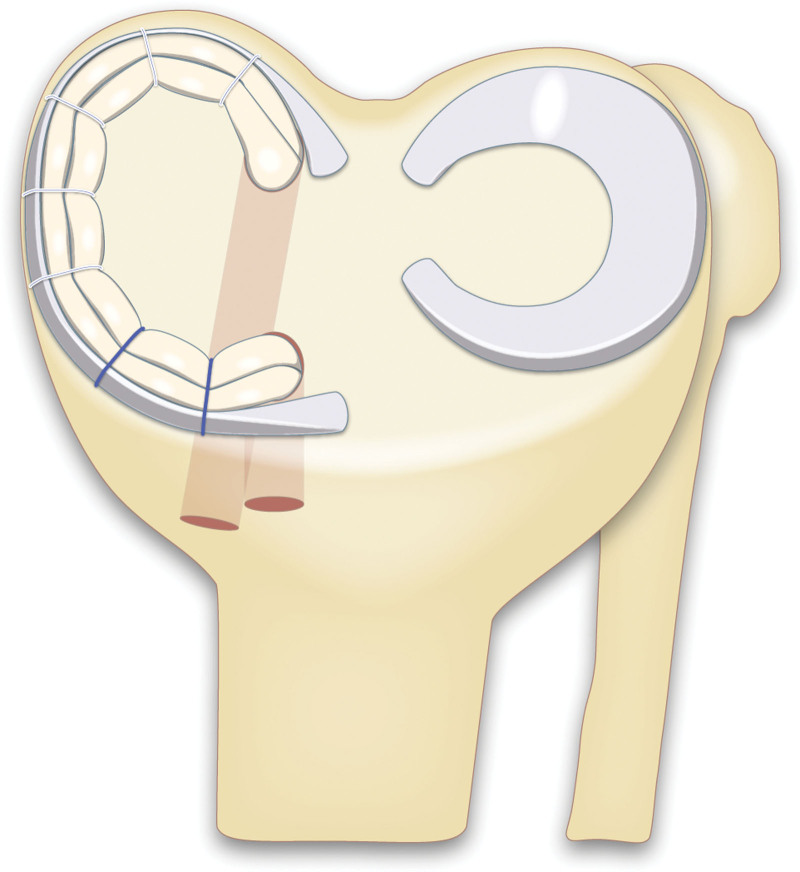
Medial meniscus reconstruction using tendon autograft.

All surgeries were performed by the same surgical team. Subtotal or total meniscectomy was performed. A tendon autograft was harvested and prepared. The anterior and posterior tunnels for medial meniscus reconstruction were drilled. The anterior and posterior ends of the prepared tendon were pulled into the tibial tunnels. The midbody and posterior horn of the intra-articular graft were sutured using the all-inside method, and the anterior horn was sutured using the outside-in method. Finally, with the graft tension appropriately set, the anterior and posterior intratunnel grafts were fixed (Fig. 1).^[18,20]^

### 2.4. Imaging

Images were acquired at Dalian University Affiliated Xinhua Hospital, Dalian, Liaoning, China. Fat-suppressed proton-density weighted turbo spin-echo coronal and sagittal MR images and fat-suppressed proton-density weighted BLADE axial MR images were acquired using MR VB17A (Siemens) with a magnetic field strength of 1.5 T. Radiographs were acquired using CXDI NE 213014 (Canon).^[18]^

### 2.5. Imaging analysis

Image analysis was performed by a trained researcher with 2 years of experience, blinded to all patient information not related to this study. The preoperative radiographs were used to measure variables related to bone structure. The MR images acquired at 1 week and 1 year postoperatively were used to measure variables related to the meniscus. The signal intensity and area measurements were recorded to a precision of 0.001. The length measurements were recorded to a precision of at least 1 pixel. The angle measurements were recorded to a precision of 0.01.

**Figure 2.**
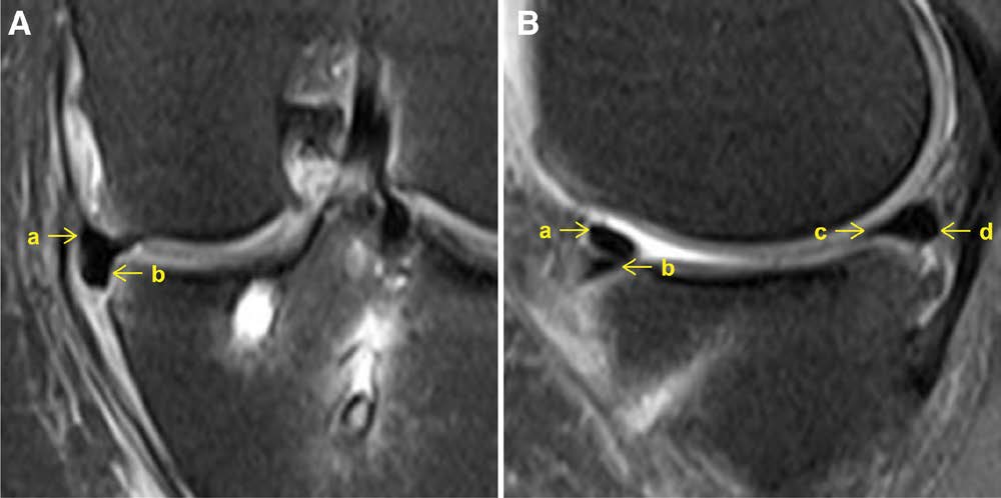
Postoperative coronal and sagittal magnetic resonance images. (A) a, remaining midbody; b, midbody tendon autograft. (B) a, anterior horn tendon autograft; b, remaining anterior horn; c, posterior horn tendon autograft; d, remaining posterior horn.

**Figure 3.**
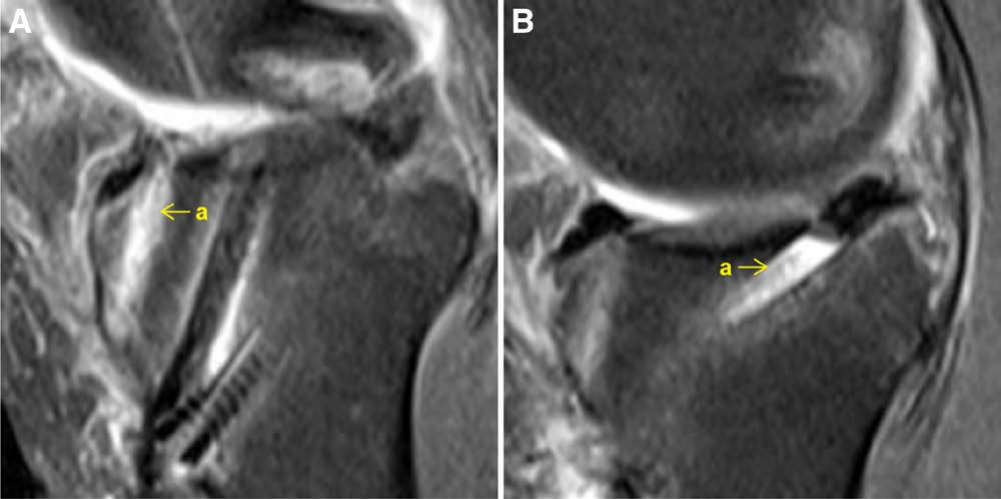
Postoperative sagittal magnetic resonance images. (A) a, anterior intratunnel tendon autograft. (B) a, posterior intratunnel tendon autograft.

Coronal MR images with the narrowest joint space width were used to measure the midbody SNR, remaining midbody cross-sectional area, and medial extrusion. Sagittal MR images with the narrowest joint space width were used to measure the anterior and posterior horn SNRs, remaining anterior and posterior horn cross-sectional areas, and anterior and posterior extrusions (Fig. 2). Sagittal MR images with the widest portion of the anterior or posterior bone tunnel near the intra-articular aperture were used to measure the anterior or posterior intratunnel SNR (Fig. 3).^[18]^

The graft SNR was calculated by dividing the signal intensity of the entire tendon autograft by the signal intensity of a large uniform background noise area medial or anterior to the knee. The background area was selected anterior to the knee on sagittal images and medial to the knee on coronal images.^[21]^


$$\begin{array}{l} Graft\;SNR=\frac{The\;signal\;intensity\;of\;the\;tendon\;autograft}{\begin{array}{ll} The\;signal\;intensity\;of\;a\;uniform\;background\; & noise\;area\;medial\;or\;anterior\;to\;the\;knee\; \end{array}} \end{array}$$

The mean SNR was the arithmetic mean of the midbody, anterior horn, posterior horn, anterior intratunnel, and posterior intratunnel SNRs.

**Figure 4.**
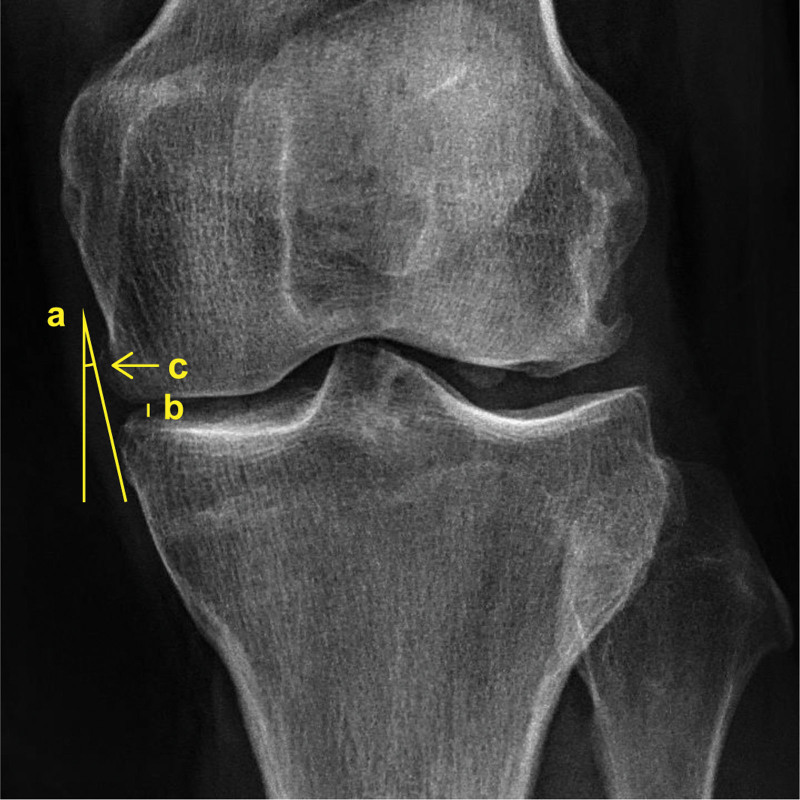
Preoperative anteroposterior knee radiograph. a, medial edge incline angle: the angle between the common tangent to the medial edges of the femur and tibia and the vertical line; b, medial joint space: the length of the vertical line segment from the medial edge of the tibia plateau to the femoral condyle; c, osteophyte on the medial edge of the femoral condyle.

The following radiographic parameters were evaluated: Kellgren–Lawrence grade, hip–knee–ankle angle, bilateral hip–knee–ankle angle difference, absolute joint space width, relative joint space width, medial edge incline angle, bilateral medial edge incline angle difference, medial posterior tibial slope, medial extrusion, anterior extrusion, posterior extrusion, anterior tunnel edge distance, and posterior tunnel edge distance (Fig. 4).^[18,22]^

### 2.6. Statistical analysis

Statistical analyses were performed using R 4.4.1 (R Foundation for Statistical Computing, Vienna, Austria). For demographic and imaging information, the mean and standard deviation were calculated for measurement data, and the number of each category was calculated for enumeration and ranked data. The paired *t* test was used to compare graft SNRs at 1 week postoperatively and at 1 year postoperatively. The Pearson correlation was used to analyze the associations between graft SNRs and potential factors. Only correlates that existed before the MRI used to measure and calculate graft SNRs were selected for regression. The ordinary least squares linear regression was used to analyze the associations between graft SNRs at 1 year postoperatively and potential factors. Multicollinearity among the predictors was assessed using the variance inflation factor. All *P* values were two-tailed. A *P* value < .05 was considered statistically significant.

## 3. Results

### 3.1. Demographic and imaging information

The midbody SNR significantly increased from 1.685 ± 0.921 at 1 week postoperatively to 12.023 ± 8.238 at 1 year postoperatively (*P* = .003). The anterior horn SNR significantly increased from 3.187 ± 1.222 at 1 week postoperatively to 19.242 ± 5.804 at 1 year postoperatively (*P* < .001). The posterior horn SNR significantly increased from 2.962 ± 1.148 at 1 week postoperatively to 16.474 ± 8.355 at 1 year postoperatively (*P* = .001). The anterior intratunnel SNR did not change significantly from 16.895 ± 12.757 at 1 week postoperatively to 12.497 ± 7.364 at 1 year postoperatively (*P* = .141). The posterior intratunnel SNR did not change significantly from 19.138 ± 16.426 at 1 week postoperatively to 19.822 ± 5.360 at 1 year postoperatively (*P* = .848). The mean SNR significantly increased from 8.774 ± 5.498 at 1 week postoperatively to 16.012 ± 4.901 at 1 year postoperatively (*P* = .004; Table 1).

**Table 1:** Demographic and imaging information.

Variable	Value
Preoperative	
Age (yr)	36.000 ± 4.733
Sex, male/female	7/4
Body mass index	27.692 ± 4.979
Kellgren–Lawrence grade, grade 0/grade 1/grade 2/grade 3	2/3/3/3
Hip–knee–ankle angle (°)	175.345 ± 3.666
Bilateral hip–knee–ankle angle difference (°)	2.381 ± 2.964
Absolute joint space width (mm)	3.122 ± 0.736
Relative joint space width	0.679 ± 0.117
Medial edge incline angle (°)	6.656 ± 9.182
Bilateral medial edge incline angle difference (°)	5.104 ± 7.505
Medial posterior tibial slope (°)	15.063 ± 2.854
1 wk postoperatively	
Midbody SNR	1.685 ± 0.921
Anterior horn SNR	3.187 ± 1.222
Posterior horn SNR	2.962 ± 1.148
Anterior intratunnel SNR	16.895 ± 12.757
Posterior intratunnel SNR	19.138 ± 16.426
Mean SNR	8.774 ± 5.498
Medial extrusion (mm)	0.883 ± 1.841
Anterior extrusion (mm)	−3.683 ± 2.295
Posterior extrusion (mm)	0.276 ± 2.787
Anterior tunnel edge distance (mm)	6.775 ± 4.029
Posterior tunnel edge distance (mm)	11.180 ± 5.835
Remaining midbody cross-sectional area (mm^2^)	7.198 ± 7.468
Remaining anterior horn cross-sectional area (mm^2^)	16.357 ± 7.514
Remaining posterior horn cross-sectional area (mm^2^)	7.720 ± 5.725
1 yr postoperatively	
Midbody SNR	12.023 ± 8.238
Anterior horn SNR	19.242 ± 5.804
Posterior horn SNR	16.474 ± 8.355
Anterior intratunnel SNR	12.497 ± 7.364
Posterior intratunnel SNR	19.822 ± 5.360
Mean SNR	16.012 ± 4.901
Medial extrusion (mm)	1.624 ± 2.129
Anterior extrusion (mm)	−2.119 ± 2.533
Posterior extrusion (mm)	2.352 ± 3.804

Measurement data are given as mean ± standard deviation. Enumeration and ranked data are given as numbers for each portion.

SNR = signal-to-noise ratio.

### 3.2. Correlates of graft SNRs

The midbody SNR at 1 week postoperatively was significantly negatively correlated with body mass index (BMI; *r* = −0.704; *P* = .016) and hip–knee–ankle angle (*r* = −0.673; *P* = .023), and significantly positively correlated with the bilateral hip–knee–ankle angle difference (*r* = 0.805; *P* = .003). The midbody SNR at 1 year postoperatively was significantly negatively correlated with BMI (*r* = −0.783; *P* = .013) and significantly positively correlated with the bilateral hip–knee–ankle angle difference (*r* = 0.727; *P* = .026; Table 2).

**Table 2:** Correlation between graft SNRs and potential factors.

	Midbody SNR		Anterior horn SNR		Posterior horn SNR		Anterior intratunnel SNR		Posterior intratunnel SNR		Mean SNR	
	*r* (95% CI)	*P*	*r* (95% CI)	*P*	*r* (95% CI)	*P*	*r* (95% CI)	*P*	*r* (95% CI)	*P*	*r* (95% CI)	*P*
1 wk												
Body mass index	−0.704 (−0.917, −0.181)	**.016**	−0.264 (−0.746, 0.399)	.433	−0.305 (−0.784, 0.401)	.391	−0.214 (−0.743, 0.481)	.554	−0.084 (−0.678, 0.576)	.818	−0.259 (−0.743, 0.404)	.443
Kellgren–Lawrence grade	0.456 (−0.198, 0.829)	.158	−0.156 (−0.691, 0.490)	.647	−0.313 (−0.768, 0.354)	.349	0.826 (0.448, 0.953)	**.002**	0.649 (0.080, 0.899)	**.031**	0.766 (0.308, 0.936)	**.006**
Hip–knee–ankle angle	−0.673 (−0.907, −0.123)	**.023**	−0.481 (−0.839, 0.168)	.135	−0.166 (−0.696, 0.482)	.626	−0.448 (−0.826, 0.207)	.167	−0.153 (−0.689, 0.492)	.654	−0.350 (−0.785, 0.316)	.291
Bilateral hip–knee–ankle angle difference	0.805 (0.397, 0.947)	**.003**	0.238 (−0.423, 0.733)	.482	−0.202 (−0.715, 0.453)	.551	0.702 (0.177, 0.916)	**.016**	0.406 (−0.256, 0.809)	.215	0.598 (−0.003, 0.882)	.052
Absolute joint space width	−0.538 (−0.860, 0.091)	.088	−0.284 (−0.755, 0.381)	.397	−0.017 (−0.611, 0.589)	.959	−0.473 (−0.836, 0.177)	.142	−0.183 (−0.705, 0.468)	.591	−0.360 (−0.789, 0.306)	.277
Relative joint space width	−0.540 (−0.861, 0.088)	.086	−0.387 (−0.801, 0.277)	.240	0.067 (−0.555, 0.641)	.846	−0.710 (−0.919, −0.192)	**.014**	−0.311 (−0.768, 0.355)	.352	−0.548 (−0.864, 0.077)	.081
Medial edge incline angle	0.503 (−0.138, 0.847)	.865	0.137 (−0.504, 0.681)	.687	−0.116 (−0.669, 0.520)	.734	0.304 (−0.362, 0.764)	.364	0.153 (−0.492, 0.690)	.653	0.236 (−0.424, 0.732)	.486
Bilateral medial edge incline angle difference	0.503 (−0.138, 0.847)	.114	0.195 (−0.459, 0.711)	.567	−0.194 (−0.711, 0.459)	.568	0.633 (0.054, 0.894)	**.036**	0.341 (−0.326, 0.781)	.305	0.515 (−0.123, 0.852)	.105
Medial posterior tibial slope	0.174 (−0.605, 0.783)	.681	0.158 (−0.615, 0.776)	.708	−0.011 (−0.710, 0.699)	.980	0.695 (−0.019, 0.939)	.056	0.277 (−0.532, 0.821)	.507	0.489 (−0.329, 0.888)	.219
Medial extrusion	0.533 (−0.098, 0.858)	.091										
Anterior extrusion			0.517 (−0.120, 0.852)	.103								
Posterior extrusion					−0.240 (−0.734, 0.420)	.477						
Anterior tunnel edge distance			−0.570 (−0.872, 0.045)	.067			0.015 (−0.590, 0.609)	.965				
Posterior tunnel edge distance					−0.039 (−0.624, 0.574)	.910			−0.330 (−0.776, 0.337)	.322		
Remaining midbody cross-sectional area	0.284 (−0.381, 0.755)	.398										
Remaining anterior horn cross-sectional area			0.306 (−0.360, 0.766)	.360								
Remaining posterior horn cross-sectional area					0.356 (−0.310, 0.788)	.283						
1 yr												
Body mass index	−0.783 (−0.952, −0.248)	**.013**	−0.051 (−0.692, 0.635)	.896	0.010 (−0.700, 0.710)	.981	−0.661 (−0.921, 0.005)	.053	−0.575 (−0.897, 0.145)	.106	−0.556 (−0.891, 0.171)	.120
Kellgren–Lawrence grade	0.289 (−0.464, 0.800)	.451	0.591 (−0.121, 0.901)	.094	0.305 (−0.450, 0.806)	.425	0.830 (0.368, 0.963)	**.006**	0.754 (0.180, 0.945)	**.019**	0.755 (0.183, 0.945)	**.019**
Hip–knee–ankle angle	−0.597 (−0.903, 0.111)	.090	−0.561 (−0.892, 0.165)	.116	−0.199 (−0.762, 0.536)	.609	−0.716 (−0.935, −0.098)	**.030**	−0.693 (−0.929, −0.053)	**.039**	−0.777 (−0.951, −0.234)	**.014**
Bilateral hip–knee–ankle angle difference	0.727 (0.122, 0.938)	**.026**	0.413 (−0.346, 0.845)	.270	0.053 (−0.634, 0.693)	.893	0.936 (0.720, 0.987)	**<.001**	0.904 (0.601, 0.980)	**.001**	0.839 (0.396, 0.965)	**.005**
Absolute joint space width	−0.471 (−0.865, 0.281)	.200	−0.866 (−0.971, −0.476)	**.003**	−0.686 (−0.927, −0.039)	**.042**	−0.444 (−0.856, 0.312)	0.231	−0.576 (−0.897, 0.142)	.104	−0.857 (−0.969, −0.447)	**.003**
Relative joint space width	−0.548 (−0.889, 0.182)	.127	−0.517 (−0.879, 0.224)	.154	−0.023 (−0.677, 0.651)	.953	−0.697 (−0.930, −0.062)	**.037**	−0.704 (−0.932, −0.074)	**.034**	−0.678 (−0.925, −0.025)	**.045**
Medial edge incline angle	0.224 (−0.517, 0.773)	.561	0.256 (−0.492, 0.786)	.506	−0.053 (−0.692, 0.634)	.893	0.327 (−0.431, 0.814)	.391	0.395 (−0.365, 0.839)	.292	0.303 (−0.452, 0.805)	.428
Bilateral medial edge incline angle difference	0.570 (−0.152, 0.895)	.109	0.614 (−0.084, 0.908)	.078	0.233 (−0.510, 0.777)	.546	0.712 (0.092, 0.934)	**.031**	0.795 (0.276, 0.955)	**.011**	0.804 (0.301, 0.957)	**.009**
Medial posterior tibial slope	0.202 (−0.729, 0.871)	.700	0.390 (−0.617, 0.913)	.445	−0.030 (−0.822, 0.801)	.954	0.749 (−0.159, 0.971)	.087	0.539 (−0.485, 0.940)	.270	0.434 (−0.583, 0.921)	.390
Medial extrusion	0.486 (−0.263, 0.870)	.184										
Anterior extrusion			−0.401 (−0.841, 0.359)	.285								
Posterior extrusion					0.012 (−0.657, 0.671)	.976						
Anterior tunnel edge distance			−0.123 (−0.728, 0.589)	.752			0.000 (−0.664, 0.664)	>.999				
Posterior tunnel edge distance					−0.460 (−0.861, 0.294)	.213			−0.548 (−0.889, 0.183)	.127		
Remaining midbody cross-sectional area	0.039 (−0.642, 0.685)	.920										
Remaining anterior horn cross-sectional area			0.788 (0.260, 0.953)	**.012**								
Remaining posterior horn cross-sectional area					0.456 (−0.299, 0.860)	.218						

Bold indicates *P* ≤ .05.

CI = confidence interval, SNR = signal-to-noise ratio.

The anterior horn SNR at 1 year postoperatively was significantly negatively correlated with the absolute joint space width (*r* = −0.866; *P* = .003) and significantly positively correlated with the remaining anterior horn cross-sectional area (*r* = 0.788; *P* = .012). The posterior horn SNR at 1 year postoperatively was significantly negatively correlated with the absolute joint space width (*r* = −0.686; *P* = .042; Table 2).

The anterior intratunnel SNR at 1 week postoperatively was significantly positively correlated with the Kellgren–Lawrence grade (*r* = 0.826; *P* = .002) and bilateral hip–knee–ankle angle difference (*r* = 0.702; *P* = .016), and significantly negatively correlated with the relative joint space width (*r* = −0.710; *P* = .014), and significantly positively correlated with the bilateral medial edge incline angle difference (*r* = 0.633; *P* = .036). The anterior intratunnel SNR at 1 year postoperatively was significantly positively correlated with the Kellgren–Lawrence grade (*r* = 0.830; *P* = .006) and bilateral hip–knee–ankle angle difference (*r* = 0.936; *P* < .001), and significantly negatively correlated with the hip–knee–ankle angle (*r* = −0.716; *P* = .030) and relative joint space width (*r* = −0.697; *P* = .037), and significantly positively correlated with the bilateral medial edge incline angle difference (*r* = 0.712; *P* = .031).

The posterior intratunnel SNR at 1 week postoperatively was significantly positively correlated with the Kellgren–Lawrence grade (*r* = 0.649; *P* = .031). The posterior intratunnel SNR at 1 year postoperatively was significantly positively correlated with the Kellgren–Lawrence grade (*r* = 0.754; *P* = .019) and bilateral hip–knee–ankle angle difference (*r* = 0.904; *P* = .001), and significantly negatively correlated with the hip–knee–ankle angle (*r* = −0.693; *P* = .039) and relative joint space width (*r* = −0.704; *P* = .034), and significantly positively correlated with the bilateral medial edge incline angle difference (*r* = 0.795; *P* = .011; Table 2).

The mean SNR at 1 week postoperatively was significantly positively correlated with the Kellgren–Lawrence grade (*r* = 0.766; *P* = .006). The mean SNR at 1 year postoperatively was significantly positively correlated with the Kellgren–Lawrence grade (*r* = 0.755; *P* = .019), bilateral hip–knee–ankle angle difference (*r* = 0.839; *P* = .005), and bilateral medial edge incline angle difference (*r* = 0.804; *P* = .009), and significantly negatively correlated with the hip–knee–ankle angle (*r* = −0.777; *P* = .014), absolute joint space width (*r* = −0.857; *P* = .003), and relative joint space width (*r* = −0.678; *P* = .045; Table 2).

The SNRs were not significantly correlated with the medial edge incline angle, medial posterior tibial slope, medial extrusion, anterior extrusion, posterior extrusion, anterior tunnel edge distance, posterior tunnel edge distance, remaining midbody cross-sectional area, and remaining posterior horn cross-sectional area (Table 2).

### 3.3. Predictors of graft SNRs

BMI (*b* = −1.458; *P* = .013) was a significant predictor of the midbody SNR at 1 year postoperatively. The absolute joint space width (*b* = −5.133; *P* = .001) and remaining anterior horn cross-sectional area (*b* = 0.349; *P* = .005) were significant predictors of the anterior horn SNR at 1 year postoperatively. The absolute joint space width (*b* = −7.953; *P* = .042) was a significant predictor of the posterior horn SNR at 1 year postoperatively. The bilateral hip–knee–ankle angle difference (*b* = 2.189; *P* < .001) was a significant predictor of the anterior intratunnel SNR at 1 year postoperatively. The bilateral hip–knee–ankle angle difference (*b* = 1.539; *P* = .001) was a significant predictor of the posterior intratunnel SNR at 1 year postoperatively. The absolute joint space width (*b* = −3.912; *P* = .002) and bilateral hip–knee–ankle angle difference (*b* = 0.837; *P* = .003) were significant predictors of the mean SNR at 1 year postoperatively (Table 3).

**Table 3:** Regression between graft SNRs and selected factors.

Standardization	Intercept	Absolute joint space width		Bilateral hip–knee–ankle angle difference		Body mass index		Remaining anterior horn cross-sectional area		*P*	*R* ^2^
		Coefficient (95% CI)	*P*	Coefficient (95% CI)	*P*	Coefficient (95% CI)	*P*	Coefficient (95% CI)	*P*		
Midbody SNR											
Unstandardized	50.426					−1.458 (−2.494, −0.423)	.013			.013	0.613
Standardized						−0.783 (−1.339, −0.227)					
Anterior horn SNR											
Unstandardized	29.943	−5.133 (−7.405, −2.861)	.001					0.349 (0.148, 0.551)	.005	.000	0.938
Standardized		−0.637 (−0.919, −0.355)						0.490 (0.208, 0.772)			
Posterior horn SNR											
Unstandardized	42.076	−7.953 (−15.500, −0.405)	.042							.042	0.470
Standardized		−0.686 (−1.336, −0.035)									
Anterior intratunnel SNR											
Unstandardized	6.609			2.189 (1.455, 2.923)	<.001					<.001	0.877
Standardized				0.936 (0.622, 1.250)							
Posterior intratunnel SNR											
Unstandardized	15.683			1.539 (0.889, 2.188)	.001					.001	0.818
Standardized				0.904 (0.523, 1.286)							
Mean SNR											
Unstandardized	26.352	−3.912 (−5.796, −2.027)	.002	0.837 (0.406, 1.268)	.003					<.001	0.944
Standardized		−0.575 (−0.852, −0.298)		0.538 (0.261, 0.815)							

CI = confidence interval, SNR = signal-to-noise ratio.

## 4. Discussion

This study aimed to identify predictors of graft SNRs after meniscus reconstruction using tendon autograft. The hypothesis was supported. The most important finding of this study is that the preoperative absolute joint space width, bilateral hip–knee–ankle angle difference, BMI, and remaining anterior horn cross-sectional area at 1 week postoperatively were significant predictors of graft SNRs at 1 year postoperatively. To our knowledge, this is the first study on graft SNR after meniscus reconstruction using tendon autograft, providing information on graft remodeling, indications, contraindications, surgical technique, and outcomes. Similarly, this is the first study to use perioperative images to interpret graft SNR after meniscus replacement, providing insights into the physical and biological environment of the meniscus.

The results indicate that the graft SNR and the graft extrusion increased from 1 week to 1 year postoperatively. The increase in graft extrusion can be explained by its positional and morphological adaptations under mechanical load. However, the underlying biological mechanism of graft SNR change remains unclear. Low signal intensity of a graft on MR images is often considered to be associated with high vascularity, because rapidly flowing blood commonly presents a signal void.^[21,23]^ However, although the natural meniscus shows a distinct vascular gradient from the outer, vascularized zone to the inner, avascular zone, the signal intensity of the entire meniscus is similarly low.^[1]^ This discrepancy suggests that vascularity alone is insufficient to explain the SNR variation in this study. Acute calcific tendinitis of the gluteus medius is usually accompanied by edema and effusion.^[24]^ The increased water content can cause high signal intensity on MR images.^[25]^ A native tendon in its original location bears little compression or friction. However, after meniscus reconstruction using tendon autograft, the intra-articular tendon autograft bears significant compression, and the intratunnel tendon autograft bears significant friction.^[1,26]^ A study found that dynamic disc compression increases inflammatory mediators in rat discs.^[27]^ Another study found that frictional shear stress increases inflammatory responses in human corneal epithelial cells.^[28]^ Therefore, an inflammatory response may better explain the SNR variation in this study.

The results indicate that the absolute joint space width was negatively correlated with the anterior horn, posterior horn, and mean SNRs at 1 year postoperatively. Similarly, the relative joint space width was negatively correlated with the anterior intratunnel SNR at 1 week postoperatively, and with the anterior intratunnel, posterior intratunnel, and mean SNRs at 1 year postoperatively. For the intra-articular graft, the joint space width may determine the degree of compression exerted by the tibia and femur. Knee joint space narrowing is commonly caused by articular cartilage loss, meniscus deficiency, osteophyte formation, and malalignment.^[29–31]^ Considering that the tendon autograft used to reconstruct the meniscus requires at least several months to develop adequate compressive properties, it cannot fully correct joint space narrowing.^[18]^ Similarly, it cannot fully correct malalignment by correcting joint space narrowing. Additionally, the tendon autograft cannot directly repair the articular cartilage or remove osteophytes. Therefore, theoretically, a narrower joint space could increase compression on the intra-articular graft, which in turn could increase tension on the intratunnel graft. Therefore, a smaller joint space may be associated with a higher graft SNR.

The results indicate that the hip–knee–ankle angle was negatively correlated with the midbody SNR at 1 week postoperatively, and with the anterior horn, posterior horn, and mean SNRs at 1 year postoperatively. Similarly, the bilateral hip–knee–ankle angle difference was positively correlated with the midbody and anterior intratunnel SNRs at 1 week postoperatively, and with the midbody, anterior intratunnel, posterior intratunnel, and mean SNRs at 1 year postoperatively. The hip–knee–ankle angle reflects lower limb alignment. Because of altered biomechanical torque, a smaller hip–knee–ankle angle increases medial tibiofemoral compression, which in turn increases tension on the intratunnel graft.^[32]^ Therefore, a smaller hip–knee–ankle angle may be associated with a higher graft SNR.

The results indicate that the bilateral medial edge incline angle difference was positively correlated with the anterior intratunnel SNR at 1 week postoperatively, and with the anterior intratunnel, posterior intratunnel, and mean SNRs at 1 year postoperatively. The medial edge incline angle difference reflects the relative position between the medial edges of the femoral condyle and the tibial plateau. Considering that the native meniscus is attached to the tibial plateau and that the joint space is widened during arthroscopy, the tendon autograft is usually positioned based on the tibial plateau. However, the femoral condyle inevitably compresses the intra-articular graft postoperatively.^[33]^ Theoretically, medial femoral translation increases the bilateral medial edge incline angle difference, which in turn increases compression on the intra-articular graft and tension on the intratunnel graft.^[18]^ An osteophyte on the medial edge of the femoral condyle can also increase the bilateral medial edge incline angle difference (Fig. 4). The compression from the osteophyte on the medial edge of the femoral condyle to the graft varies during knee motion.^[34]^ This causes unstable tension on the intratunnel graft, which can in turn cause friction between the intratunnel graft and the bone tunnel. Therefore, a larger bilateral medial edge incline angle difference may be associated with a higher graft SNR.

The results indicate that the Kellgren–Lawrence grade was positively correlated with the anterior intratunnel, posterior intratunnel, and mean SNRs at 1 week and 1 year postoperatively. The Kellgren–Lawrence grade is commonly used to assess knee osteoarthritis and is primarily based on joint space narrowing and osteophyte formation.^[22]^ Our findings indicate that joint space narrowing and osteophyte formation may increase graft SNR. Therefore, a higher Kellgren–Lawrence grade may be associated with a higher graft SNR.

The results indicate that the remaining anterior horn cross-sectional area was positively correlated with the anterior horn SNR at 1 year postoperatively. After meniscectomy, the anterior horn more commonly remains than the midbody or posterior horn. The meniscal blood supply derives from the synovium and decreases from the outer zone to the inner zone.^[1]^ Considering that meniscectomy typically excises the meniscus from the inner zone to the outer zone, a larger remaining meniscus area suggests that a greater proportion of the graft is connected to a less vascularized zone. Adequate blood supply is crucial for promoting the resolution of inflammation.^[35–37]^ Additionally, a larger remaining meniscus could increase compression on the intra-articular graft. Therefore, larger remaining anterior horn cross-sectional areas may be associated with a higher graft SNR.

The results indicate that BMI was significantly correlated with the midbody graft SNR at 1 week and 1 year postoperatively. Theoretically, a higher BMI may be associated with greater midbody pressure. Therefore, a higher BMI may lead to a higher midbody SNR. Similarly, a study reported that BMI was significantly negatively correlated with the decrease in SNR of the repaired posterior roots from 3 months to 3 years postoperatively after pullout repair for medial meniscus posterior root tears.^[38]^

This study had strengths. First, our use of a longitudinal study design ensured that all predictors of graft SNRs at 1 year postoperatively existed before the MRI used for measuring and calculating graft SNRs, whereas a cross-sectional study design can only investigate factors existing at the same time. Considering that the cause precedes the effect, this study provides stronger evidence for potential causal relationships. Second, this study not only identified many correlates for all graft portions but also constructed interpretable regression models with high goodness of fit.

This study had limitations. First, the sample size was small, largely because of the inherent rarity of meniscus reconstruction using tendon autograft.^[10]^ A small sample size increases the risk of selection bias and variability. A small sample size also limits the statistical power. If the sample size had been larger, this study would have had greater power to identify potential correlates and predictors of graft SNRs. Therefore, this study is exploratory. The findings are preliminary and hypothesis-generating rather than conclusive. They should be interpreted with caution. Considering the small sample size, future larger and prospective studies are necessary to validate these preliminary findings. Second, the data in this study were acquired from a single hospital using a single type of meniscus surgery in a homogeneous group of young patients. Therefore, the findings may not be directly generalizable to other patient populations, surgical techniques, or clinical settings. Third, this study did not investigate some other potential correlates and predictors of graft SNRs, such as patient activity level, rehabilitation protocol, and biomarkers. Fourth, BMI is influenced by fat mass and fat-free mass. This study did not differentiate body composition. Further studies are needed to clarify the relationship between leg fat mass and fat-free mass and SNR.

## 5. Conclusion

The results indicate that the preoperative absolute joint space width, bilateral hip–knee–ankle angle difference, BMI, and remaining anterior horn cross-sectional area at 1 week postoperatively are significant predictors of graft SNRs at 1 year postoperatively. To our knowledge, this is the first study on graft SNR after meniscus reconstruction using tendon autograft. These preliminary findings on graft remodeling may help to improve patient selection and surgical technique for meniscus reconstruction using tendon autograft. Considering the small sample size, future larger and prospective studies are necessary to validate these preliminary findings.

## Author contributions

**Conceptualization:** Tian-Wang Zhu.

**Formal analysis:** Tian-Wang Zhu.

**Investigation:** Tian-Wang Zhu.

**Methodology:** Tian-Wang Zhu.

**Project administration:** Tian-Wang Zhu.

**Resources:** Tian-Wang Zhu, Hao Chen, Chun-Hui Li, Rui-Xin Li.

**Software:** Tian-Wang Zhu.

**Visualization:** Tian-Wang Zhu.

**Writing – original draft:** Tian-Wang Zhu.

**Writing – review & editing:** Wan-Rui Shi, Yan-Lin Li.
